# MUC1 promotes lung metastases of liver cancer by impairing anti-tumor immunity

**DOI:** 10.1007/s12672-023-00627-0

**Published:** 2023-02-04

**Authors:** Yanze Yin, Changjie Yang, Jiafeng Xu, Yi Luo, Qiang Xia, Kang He

**Affiliations:** 1 Department of Liver Surgery, School of Medicine, Renji Hospital, Shanghai Jiao Tong University, Shanghai, China; 2 Shanghai Engineering Research Center of Transplantation and Immunology, Shanghai, China; 3 Shanghai Institute of Transplantation, Shanghai, China; 4 School of Economics and Finance, Shanghai International Studies University, Shanghai, China

**Keywords:** Liver cancer, Lung metastasis, CD8+T, PDL1, Metastatic microenvironment

## Abstract

**Purpose:**

MUC1 is a membrane bound protein that can regulate tumor progression but its role in tumor metastasis and the metastatic microenvironment remains unclear.

**Methods:**

We performed differential gene analysis for primary liver cancer (n = 31) and lung metastases (n = 31) using the Gene Expression Omnibus (GEO) dataset (GSE141016) and obtained RNA sequencing data from 374 liver cancer and 50 normal tissues from The Cancer Genome Atlas (TCGA). We analyzed the prognostic value of MUC1 and the relationship between MUC1 and the TME using online databases and a clinical cohort. Immunohistochemistry detected MUC1 in normal liver, liver cancer, and lung metastases. Multiplex immunohistochemistry staining detected immune cells in the metastatic microenvironment.

**Results:**

High MUC1 expression levels in hepatocellular carcinoma are associated with worse clinical prognosis and higher rates of lung metastasis. In addition, we observed a correlation between MUC1 and multiple immune cells in the metastatic microenvironment. In paired primary liver cancer and lung metastatic tumor tissues from the same patient, we observed higher MUC1 protein levels in lung metastases than in primary liver cancer. Furthermore, MUC1 was negatively correlated with CD8+T and Treg cells in the metastatic tumor microenvironment and positively correlated with DC. In addition, we found that MUC1 was associated with CD8+T cell activation and function using flow cytometry in another cohort of patients with liver cancer.

**Conclusion:**

These data confirm the potential of MUC1 as a prognostic marker and therapeutic target.

**Supplementary Information:**

The online version contains supplementary material available at 10.1007/s12672-023-00627-0.

## Introduction

Hepatocellular carcinoma (HCC) is one of the most malignant cancers with high incidence and morbidity rates [1]. Despite tremendous advances in clinical treatment over the last decades, HCC remains a leading cause of cancer-related mortality worldwide; metastasis, especially lung metastasis, is largely responsible for the poor prognosis. Metastatic TME is important for the initiation of primary tumor metastasis. However, the mechanism by which tumor cells reshape the pre-metastatic microenvironment remains unclear.

The metastatic TME consists of a variety of cellular and extracellular matrix components, of which immune cells are important components [2]. Cytotoxic CD8+tumor-infiltrating lymphocytes (TILs) can effectively kill tumor cells, but inhibitory immune cells such as myeloid-derived suppressor cells (MDSC) and regulatory T cells (Tregs) may contribute to maintaining a suppressive immune microenvironment that protects tumor cells from immune destruction [3, 4]. In addition, activation of immune checkpoints, such as the PD-L1/PD1 axis (PD1 is a T-cell co-suppressor molecule and checkpoint protein), also causes loss of CD8+TILs effector function; this can lead to CD8+T cell depletion and tumor evasion of the immune system, leading to tumor progression and metastasis [5, 6]. However, little is known about how tumor cells influence CD8+TILs function in the metastatic tumor microenvironment.

Mucin 1 (MUC1) is a membrane-bound protein in the mucin family [7]. MUC1 is normally overexpressed in many types of epithelial adenocarcinomas such as lung, colon, breast, and ovarian cancers [8]. MUC1 can regulate tumor progression through a variety of mechanisms, including immune response, tumor invasion, tumor cell proliferation and apoptosis, and angiogenesis [9, 10]. MUC1 induces apoptosis of activated T cells, leading to the inhibition of human T cell proliferation and cytotoxicity [11, 12]. Moreover, MUC1 binds siglecs and blocks toll-like receptors (TLRS) on DC, promoting immature DC and thereby reducing T cell function. MUC1 interacts with neutrophils and macrophages to protect cancer cells during metastasis [13, 14]. Abnormally high expression of MUC1 on cancer cells causes physical barriers, blocking the detection of tumor-associated antigens (TAAs) and preventing immune cells from killing tumor cells [7]. However, the effects of MUC1 on the liver cancer lung metastasis have not yet been investigated.

In this study, we confirmed the clinical significance of MUC1 in patients with HCC and analyzed the relationship between MUC1 and hepatocellular carcinoma lung metastasis. We investigated the regulation of MUC1 in immune cells in the metastatic TME. We also revealed the potential mechanism by which hepatocellular carcinoma cells upregulate MUC1 and affect the function of CD8+TILs to promote hepatocellular carcinoma lung metastasis. Our study provides new therapeutic targets for HCC treatment.

## Materials and methods

### Human tissues

A tumor microarray (TMA) from HCC patients (n = 80) with follow-up information was used for analysis. Specimens of primary liver tumors and lung metastases from the same patient (n = 17) were obtained from Shanghai Renji Hospital. These were provided with informed patient consent and approval from the ethics review committees of Shanghai Renji Hospital, Shanghai Jiao Tong University.

### Online data acquisition and analyses

We performed differential gene analysis for primary liver cancer (n = 31) and lung metastases (n = 31) using the Gene Expression Omnibus (GEO) dataset (GSE141016). We obtained RNA sequencing data from 374 liver cancer and 50 normal tissues from The Cancer Genome Atlas (TCGA). Based on the expression level of MUC1, hepatocellular carcinoma patients were divided into MUC1 high and low groups, and Overall Survival (OS) and Progression Free Survival (PFS) were analyzed separately. To assess the abundance of immune cell infiltration, gene expression profiles were used to assess the abundance of immune cell infiltration in tumor using ESTIMATE, and the ESTIMATE scores, immune scores, and stromal scores were calculated. The relationship between MUC1 and immune cell gene expression was calculated using the ssGSEA algorithm. As positive gene sets, gene sets with standardized p < 0.05 and false discovery rate p < 0.05 were accepted.

### Immunohistochemistry (IHC) staining

The assay was performed according to the procedure description previously [15]. The primary anti-MUC1 (Abcam, Cambridge, UK) antibody (1:200 dilution) was used for IHC staining.

### Multiple immunohistochemistry (mIHC) staining

We performed the fluorescent dye experiments according to the procedure description previously [16] by using the CD11c, CD86, CD206, CD66b, CD14, CD11b, CD19, CD8, CD4, Foxp3, and CD56 anti-human antibodies (Abcam). Using the PerkinElmer Vectra3® platform, we scanned the slides and quantified the results.

### Flow cytometry

Flow cytometry experiments were performed according to the procedure described earlier [15]. In particular, cells suspensions were incubated with APC-Cy7 live/dead (BD, 1:100), PC5.5 anti-human CD4 (BD, 1:100), BV421 anti-human TNF-α (BD, 1:100), BV605 anti-human CD8 (BD, 1:100), BV650 anti-human IFNγ (BD, 1:100), APC anti-human IL2 (BD, 1:100), AF700 anti-human GZMB (BD, 1:100), PE anti-human CD69 (BD, 1:100), and BV510 anti-human CD3 (BD, 1:100) antibodies. Flow cytometry was performed using a BD Fortessa FACS with Diva software v6.0. and results were quantified using FlowJo V10 software.

### Statistical analyses

Analyses were performed using GraphPad Prism 7.0 (La Jolla, CA, USA). We compared the differences between the two groups using Student's t-tests. Pearson's correlation test was used to analyze correlations, and the t-test was used to perform univariate analysis. The log-rank test was used for follow-up data, and p values < 0.05 were considered statistically significant.

## Results

### MUC1 is associated with a worse clinical outcome

**Fig. 1:**
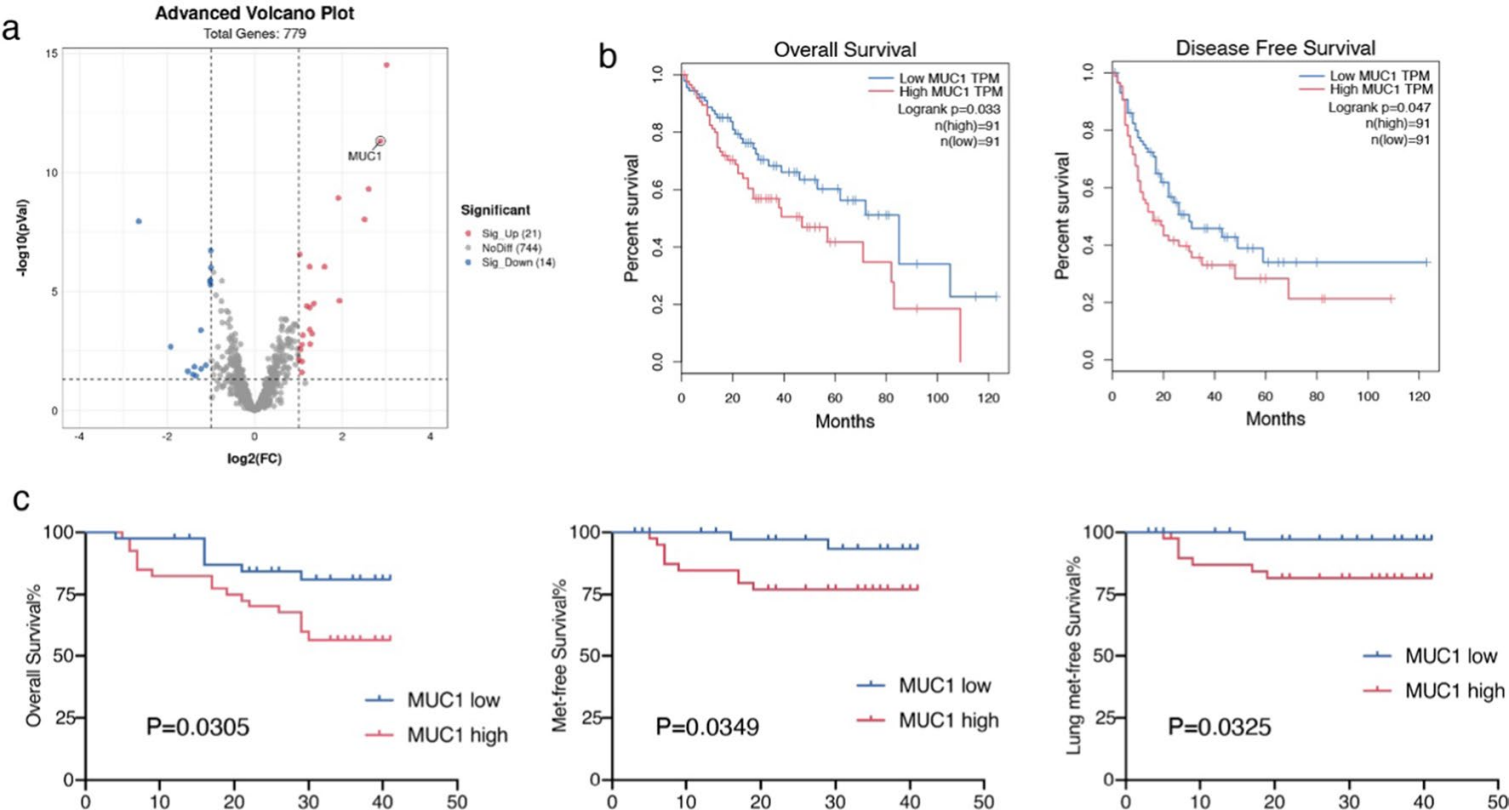
MUC1 associates with a worse clinical outcome. **a** Volcano plots of differential gene expression in 31 patients with paired primary tumors and lung metastases. Blue and red dots represent downregulated and upregulated genes, respectively. **b** MUC1 expression analysis of Overall survival and progress free interval of MUC1 high group and MUC1 low group from LIHC of The Cancer Genome Atlas (TCGA). **c** MUC1 expression analysis of Overall survival, met-free survival and lung met-free survival in a liver cancer cohort from Shanghai Renji Hospital (n = 80). *P < 0.05, **P < 0.01, ***P < 0.001, by log rank test

To identify the key genes associated with lung metastases from HCC, we performed differential gene analysis of primary liver cancer and lung metastases using the Gene Expression Omnibus (GEO) dataset (GSE141016) (Fig. 1a). The results revealed that MUC1 was expressed differentially between the two groups and prominently upregulated in lung metastases. To further investigate the reliability of the correlation between MUC1 upregulation and poor prognosis, we explored MUC1 expression levels in the liver hepatocellular carcinoma (LIHC) dataset from TCGA database and found that high MUC1 levels were related to shorter overall survival (OS) and progression-free survival (PFS) (Fig. 1b). We analyzed an HCC cohort and consistently found that patients with high MUC1 levels were prone to worse overall survival (P = 0.0305), metastasis-free survival (P = 0.0349), and lung metastasis-free survival (P = 0.0325) (Fig. 1c). In addition, the expression level of MUC1 in tumor tissue was higher than that in normal liver tissue (P = 0.0462) according to TCGA and GTEx data (Additional file 1: Supplementary Fig. 1a). However, in a clinical cohort of HCC patients, the protein levels of MUC1 were detected using immunohistochemical (IHC) staining and were also not statistically significantly different between HCC and normal liver tissue. (Additional file 1: Supplementary Fig. 1b).

### MUC1 level is significantly correlated with liver cancer lung metastasis

**Fig. 2:**
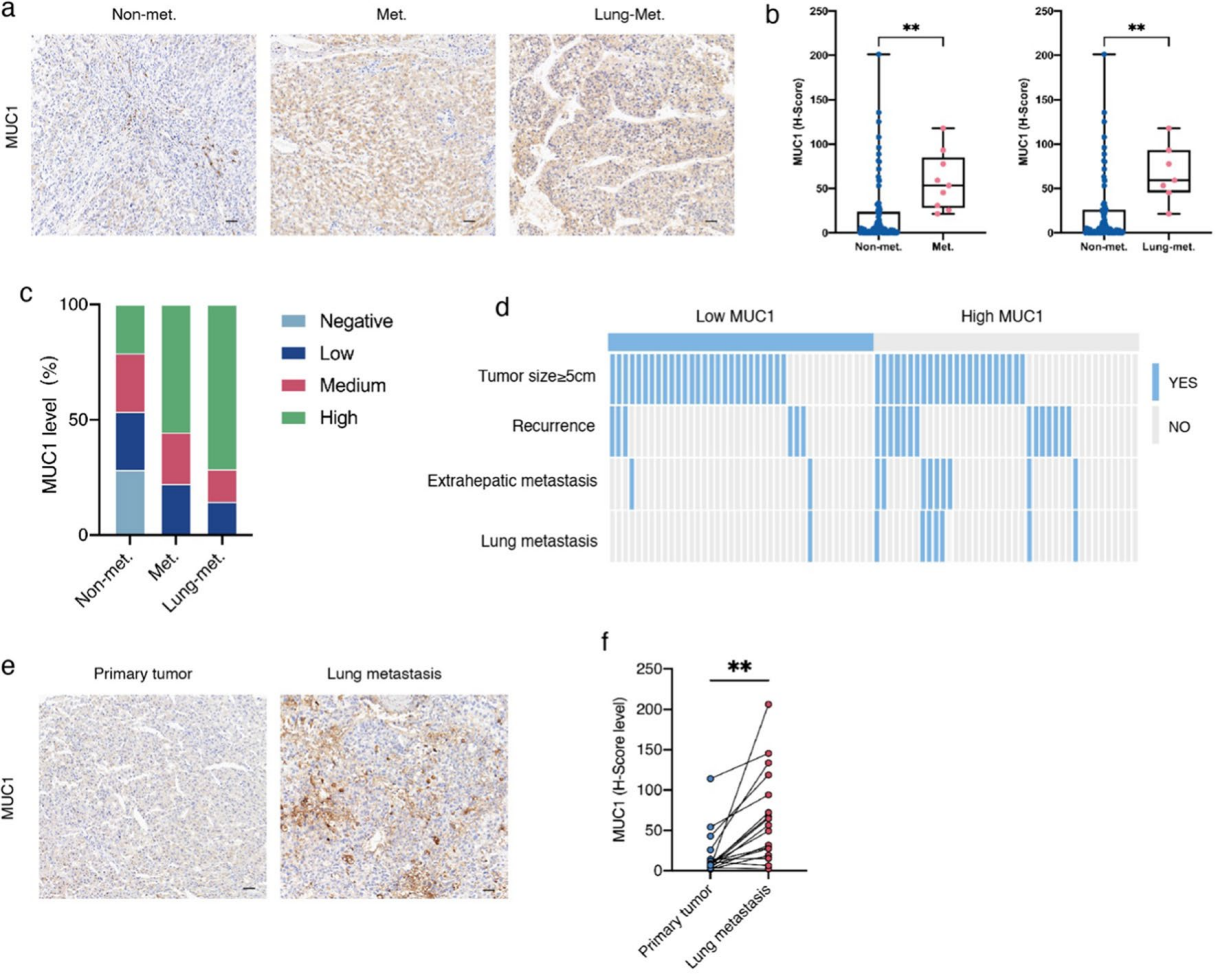
MUC1 expression is significantly correlated with liver cancer lung metastasis. **a**–**c** MUC1 expression level in non-metastasis (n = 69), extrahepatic metastasis(n = 11) and lung metastasis (n = 7). (a) Representative pictures of MUC1 immunohistochemistry staining. **b** Composition of MUC1 expression level in non-metastasis, extrahepatic metastasis and lung metastasis. **c** Comparison of MUC1 between non-metastasis and extrahepatic metastasis, non-metastasis and lung metastasis, respectively. **d** Clinical events of MUC1 high group (n = 40) and MUC1 low group (n = 40). **e**, **f** Comparison of MUC1 between paired primary tumors and lung metastases. **e** Representative pictures of MUC1 immunohistochemistry staining and **f** statistics chart. Scale bars, 50 μm. *P < 0.05, **P < 0.01, ***P < 0.001, by chi-squared test (**d**) and two-tailed unpaired (**c**) or paired (**f**) t test

To investigate the relationship between MUC1 and lung metastasis in HCC, we detected the protein levels of MUC1 in patients with no metastasis, patients with extrahepatic metastasis, and patients with lung metastasis in the HCC cohort by IHC assay. We found that MUC1 was significantly upregulated in patients with extrahepatic metastasis and patients with lung metastasis compared to patients without metastasis (Fig. 2a, b). The percentages of MUC1 high and medium groups were significantly higher in patients with extrahepatic metastasis and lung metastasis than in patients without metastasis (Fig. 2c). We found no statistically significant correlation between MUC1 expression and tumor size and recurrence rate, while extrahepatic metastasis and lung metastasis rates were higher in the MUC1 high group than in the MUC1 low group (Fig. 2d). In addition, we analyzed the MUC1 protein levels in primary neoplasms and lung metastases of HCC from the same patient by immunohistochemical staining and found that the MUC1 protein levels in lung metastases were remarkably higher than those in primary HCC (Fig. 2e, f).

### MUC1 is associated with the TME

**Fig. 3:**
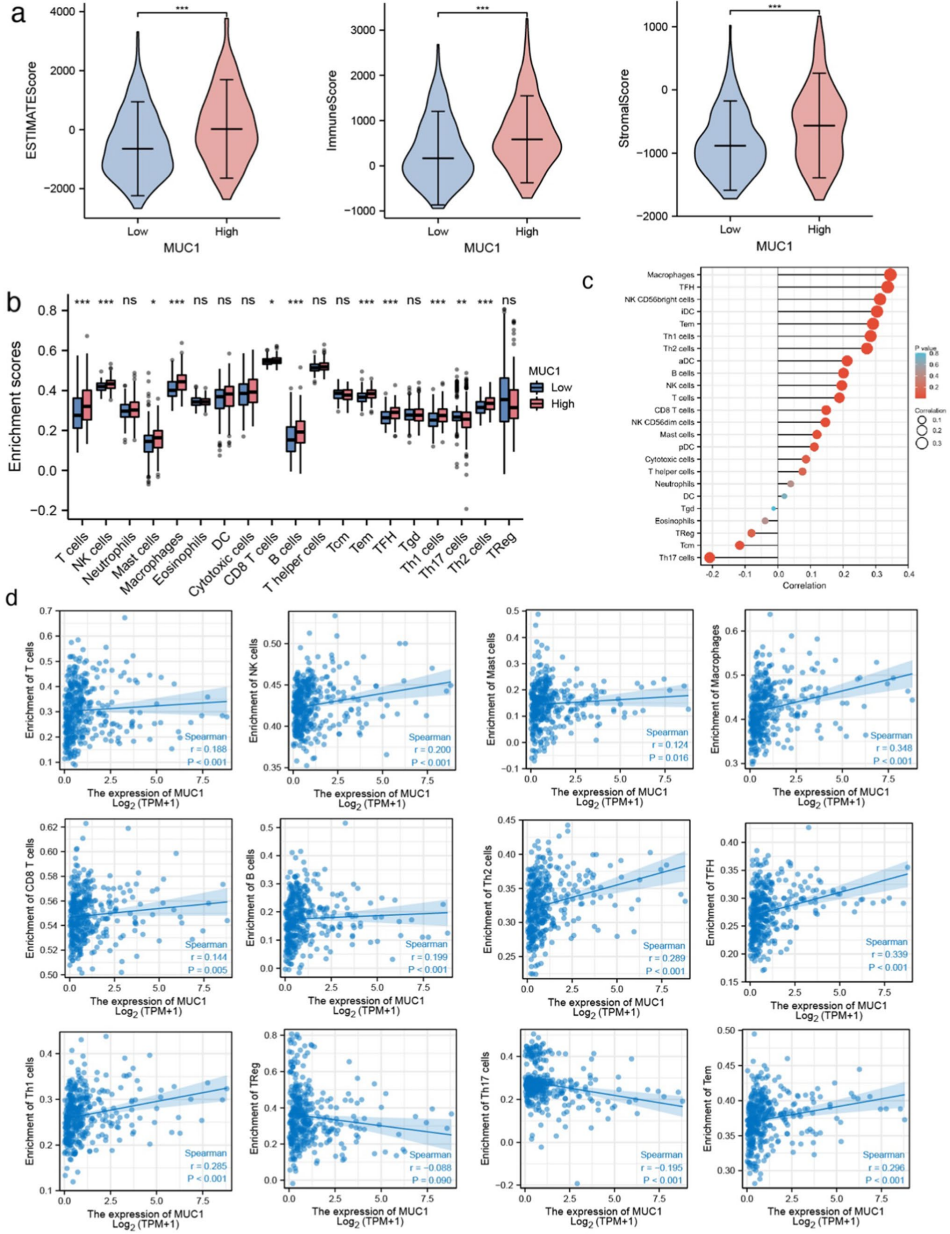
MUC1 associates with tumor immune microenvironment. **a** Violin plots show ESTIMATE score, Immune score and Stromal score of MUC1 high and low group from TCGA. (b-d) Analyses of MUC1 expression and immune infiltration in LIHC of TCGA. **b** Enrichment score of infiltration profile of immune subsets based on MUC1 expression levels from TCGA using ssGSEA. **c** Lollipop plots show correlation between MUC1 and immune infiltration in LIHC of TCGA using ssGSEA. **d** Correlation analysis of MUC1 and immune cells from TCGA using ssGSEA. *P < 0.05, **P < 0.01, ***P < 0.001, by Pearson correlation analysis

Next, to investigate whether MUC1 regulates tumor metastasis by reprogramming the immune microenvironment, we calculated the ESTIMATE score, immune score, and stromal score according to LIHC from TCGA database, and found that the ESTIMATE score, immune score, and stromal score of the MUC1 high group were higher than those of the MUC1 low group (Fig. 3a). To further explore the effect of MUC1 on the TME, we analyzed the correlation between MUC1 and immune cells using TCGA database; we found that the MUC1 high group had more T cells, NK cells, mast cells, macrophages, CD8 + T, B cells, Tem, TFH, Th1 cells, and Th2 cells and lower Th17 cells compared to the MUC1 low group (Fig. 3b). MUC1 was positively correlated with macrophages, T cells, DC, NK cells, mast cells, CD8 + T cells, B cells, Tem, TFH, Th1, and Th2 cells, and negatively correlated with Tem, Treg, and Th17 cells (Fig. 3c). Furthermore, we analyzed the data using the ssGSEA algorithm which showed that MUC1 was positively correlated with T cells (r = 0.188, p < 0.001), NK cells (r = 0.200, p < 0.001), mast cells(r = 0.124, p < 0.001), macrophages (r = 0.348, p < 0.001), CD8 + T cells (r = 0.144, p = 0.005), B cells (r = 0.199, p < 0.001), Tem (r = 0.296, p < 0.001), TFH (r = 0.339, p < 0.001), Th1 cells (r = 0.285, p < 0.001), and Th2 cells (r = 0.289, p < 0.001), and negatively correlated with Th17 (r = − 0.195, p < 0.001) cells and Tregs (r = − 0.008, p = 0, 090) (Fig. 3d and Additional file 1: Supplementary Fig. 2a).

### Association between MUC1 and TILs in lung metastases

**Fig. 4:**
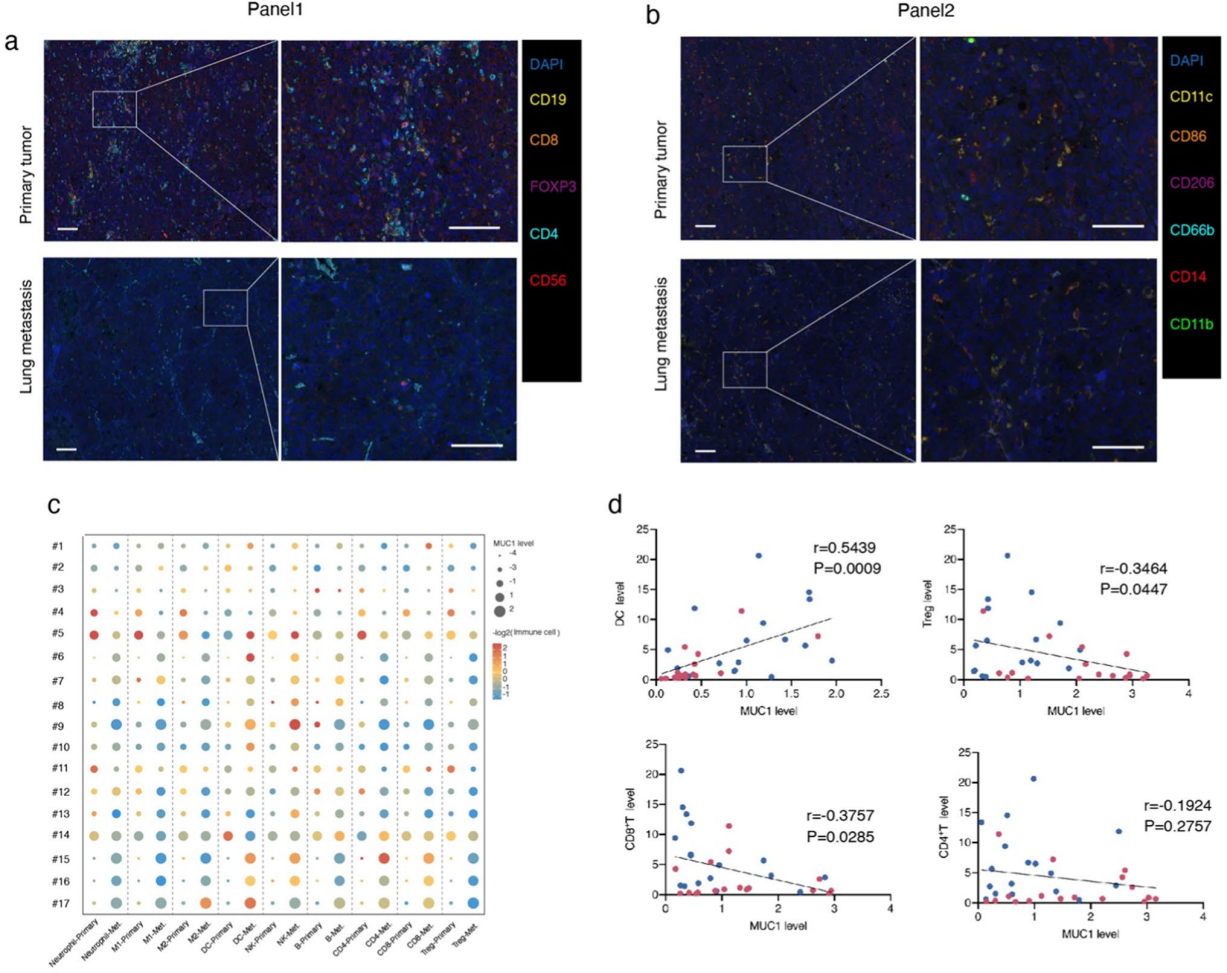
Association of MUC1 and TILs in lung metastasis. **a**–**d** Analyses of MUC1 expression and immune infiltration in paired primary tumors and lung metastases form the same patients (n = 17) by multi-immunohistochemistry (mIHC) staining of metastasis-associated lymphatic and myeloid immune cells. Representative mIHC staining images of metastasis-associated **a** lymphatic and **b** myeloid immune cells. Scale bars, 50 μm. **c** Dot plots displaying MUC1 expression level and altered immune cells in paired primary tumors and lung metastases. **d** Correlation analyses between MUC1 and metastasis-associated immune cells. *P < 0.05, **P < 0.01, ***P < 0.001, by Pearson correlation analysis

To further explore the relationship between MUC1 and immune cells in the TME, we examined metastasis-associated immune cells in paired primary tumor and lung metastasis tissues from the same patient using Fluorescent Multiplex Immunohistochemistry (Fig. 4a–c). In addition, we analyzed the correlation between MUC1 and individual immune cells and showed that MUC1 was positively correlated with DC (r = 0.5439, p = 0.0009) and negatively correlated with Tregs (r = − 0.3464, p = 0.0447) and CD8 + T cells (r = − 0.3757, p = 0.0285) (Fig. 4d and Additional file 1: Supplementary Fig. 3a).

### Correlation of MUC1 with immune checkpoints in the TME

**Fig. 5:**
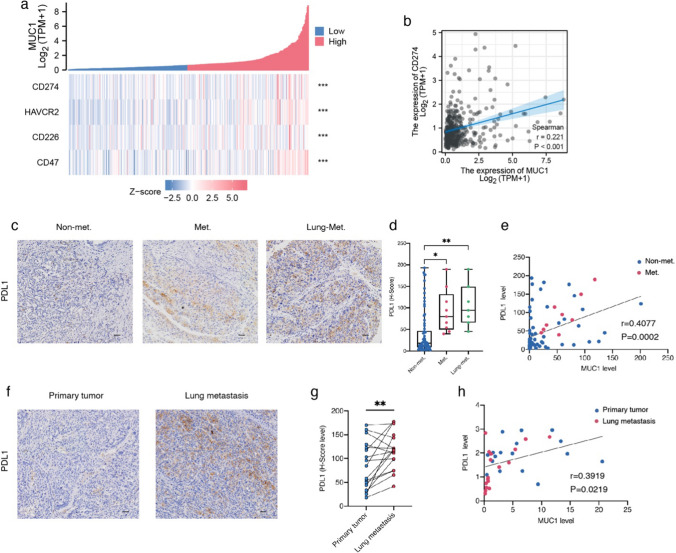
Correlation of MUC1 with immune checkpoints in tumor immune microenvironment. **a**, **b** Correlation analysis of MUC1 expression with immune checkpoints in LIHC of TCGA. **c**–**e** Analysis of MUC1 and PDL1 in HCC patients. **c** Representative pictures of PDL1 IHC staining. **d** Comparisons of PDL1 level in non-metastatic with metastatic and lung metastatic tumors. **e** Correlation analysis of MUC1 expression with PDL1 level. **f**–**h** Analysis of MUC1 and PDL1 in primary and lung metastatic tumors from the same patient. **f** Representative pictures of PDL1 IHC staining. **g** Comparisons of PDL1 level in primary and lung metastatic tumors from the same patient. **h** Correlation analysis of MUC1 expression with PDL1 level. Scale bars, 50 μm. *P < 0.05, **P < 0.01, ***P < 0.001, by two-tailed unpaired or paired t test (**d**, **g**) and Pearson correlation analysis (**b**, **e** and **h**)

To investigate the mechanism of MUC1 effect on T cells in the TME, we analyzed the relationship between MUC1 and immune checkpoints using TCGA database and found that MUC1 was positively correlated with CD274 (PDL1), HAVCR2 (TIM-3), CD226, and CD47. Among them, MUC1 was most strongly correlated with CD274 (PDL1) (r = 0.221, p < 0.001) (Fig. 5a, b). Next, we explored the relationship between MUC1 and PDL1 at the protein level. Immunohistochemical staining showed that PDL1 levels were higher in the lung metastasis group of HCC patients than in HCC patients without extrahepatic metastasis, and MUC1 was positively correlated with PDL1 (r = 0.4077, P = 0.0002) (Fig. 5c-e). In addition, we performed immunohistochemical staining of paired primary tumor and lung metastasis tissue sections from the same patient and found that PDL1 levels were higher in lung metastases than in primary tumors, and that MUC1 was positively correlated with PDL1 (r = 0.3919, P = 0.0219) (Fig. 5f-h).

### Association between MUC1 expression and CD8 + T cell function and immune checkpoints

**Fig. 6:**
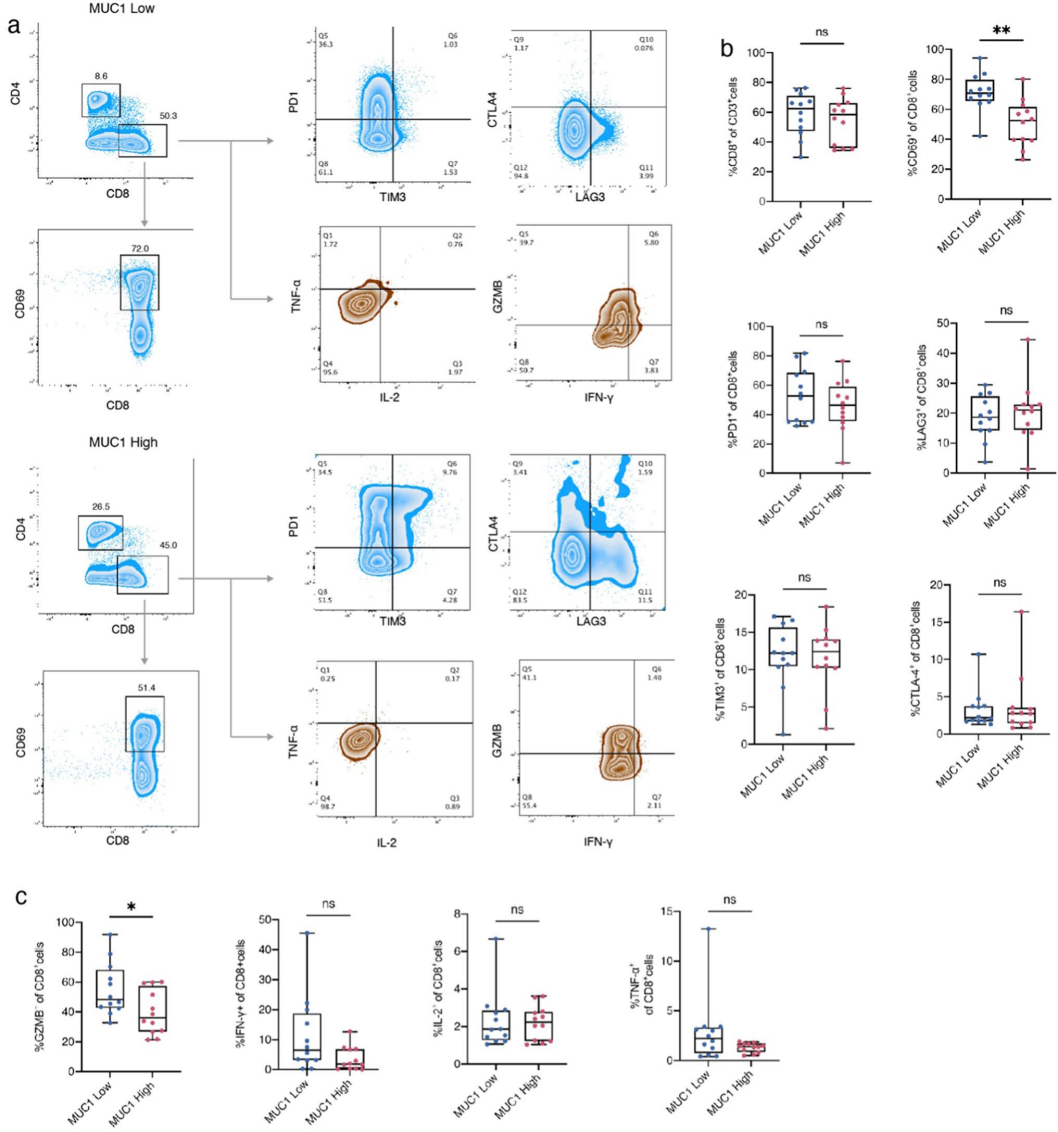
Association between MUC1 expression and CD8+T cell function and immune checkpoints. **a** Representative flow cytometry charts of CD8+T cell function and immune checkpoints based on MUC1 expression. **b** Statistical map of CD8+T cell immune checkpoints. **c** Statistical map of CD8+T cell function. *P < 0.05, **P < 0.01, ***P < 0.001, by two-tailed unpaired t test (**b**, **c**)

To further explore the relationship between MUC1 and T cells in the metastatic TME, we examined CD8+T cell function and immune checkpoints using flow cytometry in a cohort of HCC patients. The patients were divided into MUC1 high and MUC1 low groups based on MUC1 immunohistochemical staining. Figure 6a–c showed that HCC patients in the MUC1 high group had lower levels of the T cell activation marker CD69 in CD8+T cells compared to those in the MUC1 low group. Furthermore, patients in the MUC1 high group had significantly lower levels of the CD8+T function marker granzyme B than those in the MUC1 low group.

## Discussion

Tumor metastasis is a complex process that involves multiple cellular and extracellular components [17]. In addition to intrinsic tumor cell characteristics, metastatic TME influences tumor metastasis [18, 19]. Tumor cells and the metastatic TME interact and dynamically change with each other. It has been reported that primary tumors can remodel the pre-metastatic ecological niche before metastasis, making it favorable for tumor cells to metastasize [20]. We identified MUC1 expression levels were increased in hepatocellular carcinoma tissues relative to normal liver tissues from TCGA and GTEx data. However, the protein levels of MUC1 were not detected statistically significantly different in a clinical cohort of HCC patients. This may be because the sample size of the clinical cohort was not large enough. We also found that patients with high MUC1 expression levels were more likely to develop lung metastases and that MUC1 expression levels were significantly higher in lung metastases than in primary liver cancer. The reasons for the increased expression of MUC1 in HCC require further research. We analyzed the correlation between MUC1 and multiple immune cells and found that MUC1 expression was positively correlated with macrophages, T cells, DC, NK, mast cells, CD8+T, B cells, Tem, TFH, Th1 cells and Th2 cells, and negatively correlated with Th17 cells, Tem and Tregs. These findings suggest that HCC may promote lung metastasis by remodeling the metastatic microenvironment through MUC1.

The interaction between immune and tumor cells in the TME plays a key role in controlling tumor progression [21, 22]. CD8+TILs are key effector cells that directly kill tumor cells [23], and the abundance of CD8+TILs is positively correlated with clinical prognosis [24], while the T cell co-suppressor molecule PD-L1 and PD1 receptor are associated with worse clinical prognosis [25]. Recent studies have found that MUC1 is expressed on human T cells and may be involved in the regulation of T cell function [26]. A variety of factors contribute to the loss of TILs effector function, leading to tumor evasion by the immune system and tumor progression and metastasis [27, 28]. To investigate how MUC1 affects T cells in the TME. Using immunofluorescence staining of paired primary liver cancer and lung metastatic tumor tissues from the same patient, we found that MUC1 expression was negatively correlated with CD8+T and Treg cells in the metastatic TME and positively correlated with DC cells. We analyzed the relationship between MUC1 and immune checkpoints using TCGA database and found that MUC1 was positively correlated with CD274. Consistent with this, in another cohort of liver cancer patients, we used flow cytometry and found higher levels of CD69+CD8+T cells and GZMB of CD8+T in the MUC1 high group, but did not find a statistical difference in CD8+T cells which may be related to sample size. According to these results, abnormally high expression level of MUC1 in liver cancer cells may inhibit the function of CD8+TILs through the high expression of PDL1, forming an inhibitory immune microenvironment, resulting in metastasis of cancer cells. Further studies are needed to clarify whether MUC1 affects other immune cells and their roles.

In this study, we reported that high MUC1 expression levels in HCC are associated with worse clinical prognoses and higher rates of lung metastasis. Multiple immune cells were found to be associated with MUC1 expression. In paired primary liver cancer and lung metastatic tumor tissues from the same patient, we observed higher MUC1 protein levels in lung metastases than in primary liver cancer, and MUC1 was negatively correlated with CD8+T and Treg cells in the metastatic TME and positively correlated with DC. In addition, in another cohort of liver cancer patients, we found that MUC1 was associated with CD8+T cell activation and function using flow cytometry. This suggests that tumors may regulate CD8+T cell function in the metastatic TME through MUC1, thereby affecting the HCC lung metastasis process. These data confirm the potential of MUC1 as a prognostic marker and therapeutic target. This will contribute to the development of new strategies for the treatment of tumor metastasis.

## Supplementary Information

**Additional file 1: Fig S1.** MUC1 expression level in liver cancer. (A, B) MUC1 expression level analysis of HCC (A) patients from LIHC of TCGA and GTEx, and (B) patients from Shanghai Renji Hospital (n=80). Scale bars, 50 μm. **Fig S2.** MUC1 associates with tumor immune microenvironment. (A) Correlation analysis of MUC1 and immune cells from TCGA using ssGSEA. **Fig S3.** Correlation of MUC1 and immune cells in lung metastasis. (A) Correlation analyses between MUC1 and metastasis-associated immune cells.

## Data Availability

The datasets generated during and/or analysed during the current study are available from the corresponding author on reasonable request.
