# Supplementary material for: MUC1 promotes lung metastases of liver cancer by impairing anti-tumor immunity
Source: Discov Oncol. 2023 Feb 4;14:18. doi: 10.1007/s12672-023-00627-0 (PMC9899301; doi:10.1007/s12672-023-00627-0)
Supplement: Supplementary file 1 — Additional file 1: Fig S1. MUC1 expression level in liver cancer. (A, B) MUC1 expression level analysis of HCC (A) patients from LIHC of TCGA and GTEx, and (B) patients from Shanghai Renji Hospital (n=80). Scale bars, 50 μm. Fig S2. MUC1 associates with tumor immune microenvironment. (A) Correlation analysis of MUC1 and immune cells from TCGA using ssGSEA. Fig S3. Correlation of MUC1 and immune cells in lung metastasis. (A) Correlation analyses between MUC1 and metastasis-associated immune cells. [file 12672_2023_627_MOESM1_ESM.docx]

## Additional file Figures


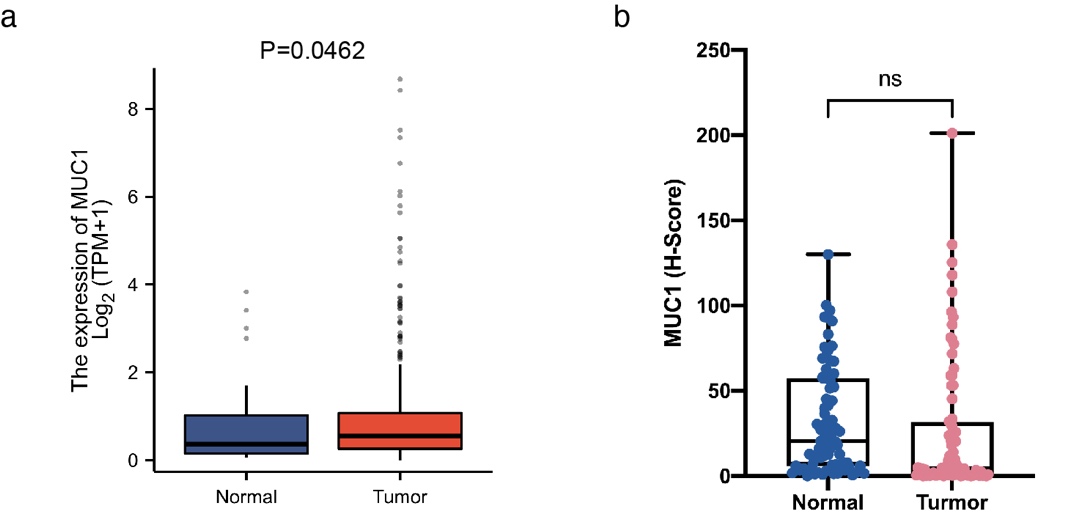


**Additional file 1: Fig S1. MUC1 expression level in liver cancer.** (A, B) MUC1 expression level analysis of HCC (A) patients from LIHC of TCGA and GTEx, and (B) patients from Shanghai Renji Hospital (n=80). Scale bars, 50 μm.


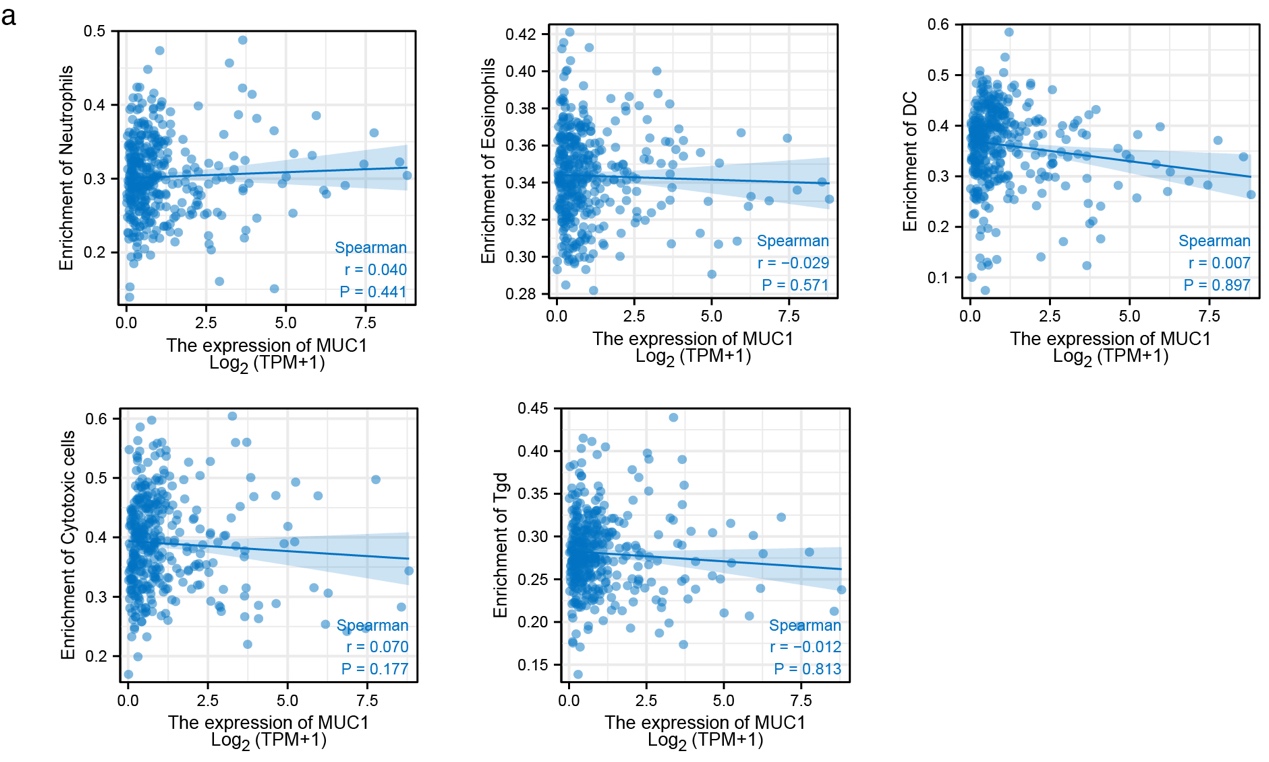


**Additional file 1: Fig S2. MUC1 associates with tumor immune microenvironment.** (A) Correlation analysis of MUC1 and immune cells from TCGA using ssGSEA.


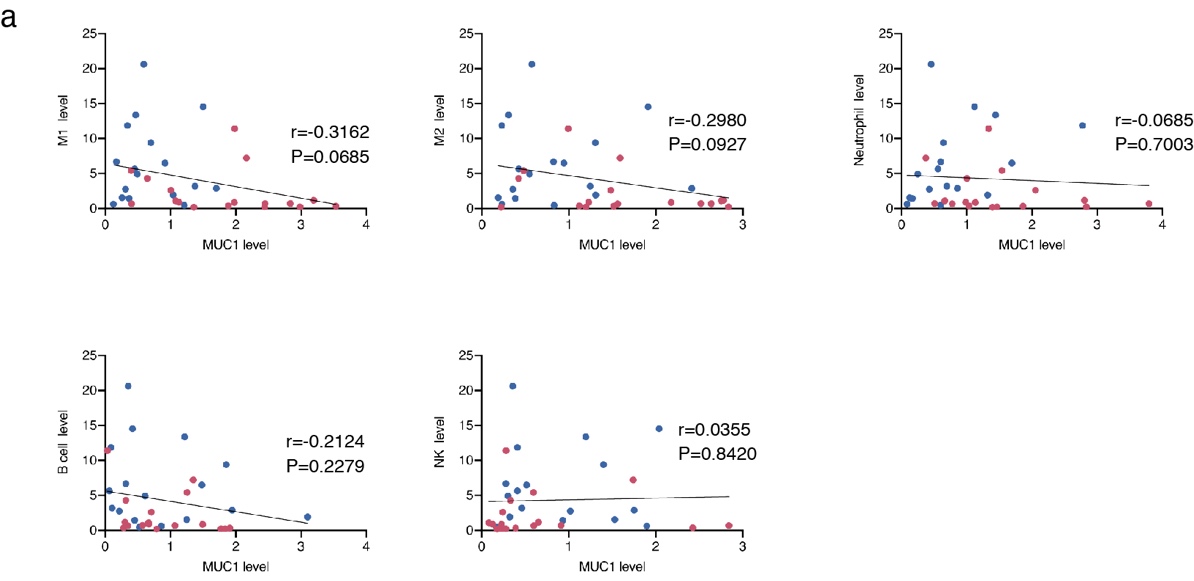


**Additional file 1: Fig S3. Correlation of MUC1 and immune cells in lung metastasis.** (A) Correlation analyses between MUC1 and metastasis-associated immune cells.
